# The complete mitochondrial genome of *Indothais lacera* (Neogastropoda: Muricidae)

**DOI:** 10.1080/23802359.2017.1407697

**Published:** 2017-11-27

**Authors:** Shengping Zhong, Yanfei Zhao, Xianfeng Wang, Zhifei Song, Qin Zhang

**Affiliations:** Key Laboratory of Marine Biotechnology, Guangxi Institute of Oceanology, Beihai, China

**Keywords:** Mitochondrial genome, *Indothais lacera*, Muricidae

## Abstract

The rock shells *Thais* is the most important genera of Muricidae. However, the systemically classification and phylogenetic studies have so far been limited. In this study, we report the complete mitochondrial genome sequence of *Indothais lacera*. The mitogenome has 15,272 base pairs (68.1% A + T content) and made up of a total of 37 genes (13 protein-coding, 22 transfer RNAs and two ribosomal RNAs), but no control region. This study was the first available complete mitogenomes of *Indothais* and will provide useful genetic information for future phylogenetic and evolutionary classification of *Thais*.

*Thais* is one of the most important genera of Muricidae, which is distributed widely in the intertidal coastal areas (Ki et al. 2010). There are various species belonging to *Thais*, however, systemically classifications on them have not been done and phylogenetic studies have so far been limited. According to the late classification, *Indothais* had been revised a new genus from *Thais* (Claremont et al. 2013). In spite of its evolutionary and ecological importance, adequate genetic information about the genus is still missing. Here, we report the first complete mitochondrial genome sequence of genus *Indothais*, which will provide a better insight into phylogenetic assessment and evolutionary classification.

A tissue sample of *I. lacera* was collected from GuangXi province, China (Beihai, 21.426659N, 109.213166E), and the whole body specimen (#GR0355) were deposited at Marine biological Herbarium, Guangxi Institute of Oceanology, Beihai, China. The total genomic DNA was extracted from the muscle of the specimens using an SQ Tissue DNA Kit (OMEGA, Guangzhou, China) following the protocol from the manufacturer. DNA libraries (350bp insert) were constructed with the TruSeq NanoTM kit (Illumina, San Diego, CA) and were sequenced (2 × 150 bp paired-end) using HiSeq platform at Novogene Company, Beijing, China. Mitogenome assembly was performed by MITObim (Hahn et al. 2013). The cytochrome oxidase subunit 1 (COI) gene of *I. lacera* (GenBank accession no., KC466631) was chosen as the initial reference sequence for MITObim assembly. Gene annotation was performed by MITOS (Bernt et al. 2013).

The complete mitogenome of *I. lacera* was 15,272 bp in length (GenBank accession no. MG099702), and containing the typical set of 13 protein-coding, 22 tRNA and two rRNA genes, but no control region. The overall base composition of the mitogenome was estimated to be A 29.5%, T 38.6%, C 15.2% and G 16.7%, with a high A + T content of 68.1%, which is similar, but slightly higher than *Thais clavigera* (66.2%) (Ki et al. 2010). The gene order in *I. lacera* is also highly similar to that found in *T. clavigera*, which indicated close relationship between *I. lacera* and *T. clavigera*. The result of phylogenetic tree of 12 species (including other 11 species from Order Neogastropoda in NCBI) also supported the close relationship between *I. lacera* and *T. clavigera* (Figure 1), as they shared the same clade with the highest bootstrap value. All protein-coding genes were found to use the initiation codon ATG except for *NAD2*, *NAD1*, and *NAD5* genes, which unlike other gastropods usually have TTG served as the initiation codon (Grande et al., 2008). The complete mitochondrial genome sequence of *I. lacera* was the first sequenced mitogenomes within the genus *Indothais*, which will contribute to further phylogenetic and comparative mitogenome studies of the genus *Indothais*, and related genera.

**Figure 1.**
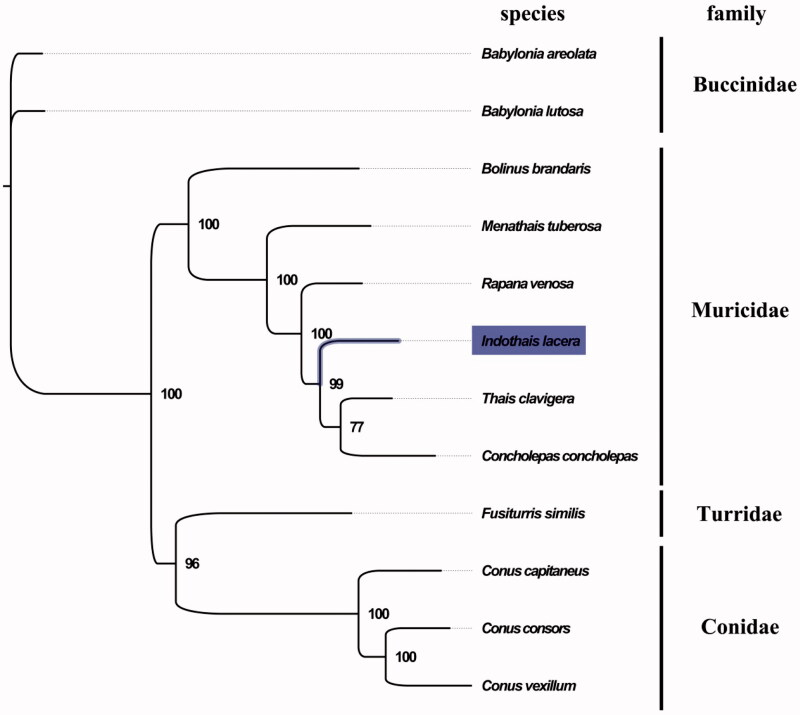
Phylogenetic tree of 12 species in order Neogastropoda. The complete mitogenomes is downloaded from GenBank and the phylogenic tree is constructed by maximum-likelihood method with 100 bootstrap replicates. The bootstrap values were labelled at each branch nodes. The gene's accession number for tree construction is listed as follows: *Babylonia areolata* (NC_023080), *Babylonia lutosa* (NC_028628), *Bolinus brandaris* (NC_013250), *Menathais tuberosa* (NC_031405), *Rapana venosa* (NC_011193), *Thais clavigera* (NC_010090), *Concholepas concholepas* (NC_017886), *Fusiturris similis* (NC_013242), *Conus vexillum* (NC_035007), *Conus consors* (NC_023460), and *Conus capitaneus* (NC_030354).
